# Toll-Like Receptor 4 Protects Against *Clostridium perfringens* Infection in Mice

**DOI:** 10.3389/fcimb.2021.633440

**Published:** 2021-03-08

**Authors:** Masaya Takehara, Keiko Kobayashi, Masahiro Nagahama

**Affiliations:** Department of Microbiology, Faculty of Pharmaceutical Sciences, Tokushima Bunri University, Yamashiro-cho, Japan

**Keywords:** TLR4, *Clostridium (C.) perfringens*, innate immunity, Granulopoiesis, G-CSF

## Abstract

Toll-like receptor 4 (TLR4) has been reported to protect against Gram-negative bacteria by acting as a pathogen recognition receptor that senses mainly lipopolysaccharide (LPS) from Gram-negative bacteria. However, the role of TLR4 in Gram-positive bacterial infection is less well understood. *Clostridium perfringens* type A is a Gram-positive bacterium that causes gas gangrene characterized by severe myonecrosis. It was previously demonstrated that *C. perfringens* θ-toxin is a TLR4 agonist, but the role of TLR4 in *C. perfringens* infection is unclear. Here, TLR4-defective C3H/HeJ mice infected with *C. perfringens* showed a remarkable decrease in survival rate, an increase in viable bacterial counts, and accelerated destruction of myofibrils at the infection site compared with wild-type C3H/HeN mice. These results demonstrate that TLR4 plays an important role in the elimination of *C. perfringens*. Remarkable increases in levels of inflammatory cytokines, such as interleukin-1β (IL-1β), interleukin-6 (IL-6), and granulocyte colony-stimulating factor (G-CSF), were observed in *C. perfringens*-infected C3H/HeN mice, whereas the increases were limited in C3H/HeJ mice. Generally, increased G-CSF accelerates granulopoiesis in the bone marrow and the spleen to exacerbate neutrophil production, resulting in elimination of bacteria. The number of neutrophils in the spleen was increased in *C. perfringens*-infected C3H/HeN mice compared with non-infected mice, while the increase was lower in *C. perfringens*-infected C3H/HeJ mice. Furthermore, DNA microarray analysis revealed that the mutation in TLR4 partially affects host gene expression during *C. perfringens* infection. Together, our results illustrate that TLR4 is crucial for the innate ability to eliminate *C. perfringens*.

## Introduction

*Clostridium perfringens* type A is a spore-forming Gram-positive anaerobic bacterium that causes gas gangrene in both humans and animals (Songer, 1996; Petit et al., 1999). The disease is a life-threatening necrotizing soft tissue infection that leads to myonecrosis, shock, multiple organ failure, and death of patients (Bryant, 2003; Titball, 2005). *C. perfringens* sepsis often develops extremely rapidly, accompanied by intravascular hemolysis and metabolic acidosis and has a high mortality rate (Hifumi, 2020). The only current therapeutic measures are hyperbaric oxygen therapy and use of antibiotics, but these treatments are not sufficient to prevent disease progression in some patients (Stephens, 1996; Bryant and Stevens, 2010). More effective therapeutic strategies are urgently required.

*C. perfringens* type A has been reported to produce multiple virulence factors. Many studies have focused on the biological and biochemical activities of *α*-toxin (or phospholipase C), which has both phospholipase C (PLC) and sphingomyelinase (SMase) activities (Bryant, 2003; Sakurai et al., 2004). Using a *C. perfringens* infection mouse model, an *α*-toxin-deficient strain was demonstrated to exhibit delayed spread of muscle necrosis (Ellemor et al., 1999). It is supposed that *α*-toxin is involved in the spread of infection by multiple mechanisms. *α*-Toxin enhances intravascular cell aggregation and impairs the host immune response by impeding inflammatory cell infiltration to the site of infection (Bryant et al., 2000; Bahl and Dürre, 2001; Ochi et al., 2002; Bryant et al., 2006). We previously reported that *α*-toxin inhibits neutrophil production by interfering with differentiation in the bone marrow, leading to the impairment of innate host immunity (Takehara et al., 2016a; Takehara et al., 2016b). These results indicate that *α*-toxin is involved in avoiding host immune clearance. Additionally, *α*-toxin induces death of endothelial cells, suggesting that it disturbs peripheral circulation (Takehara et al., 2020a). The *α*-toxin-mediated intravascular cell aggregation also promotes vascular occlusion (Bryant et al., 2000; Hickey et al., 2008). Moreover, *α*-toxin inhibits the differentiation of erythroid progenitors, which might lead to hypogloburia (Takagishi et al., 2017). These results suggest that *α*-toxin affects the tissue microcirculation, leading to ischemic conditions in the muscle tissue. Recently, we reported that *α*-toxin inhibits the differentiation of myoblasts, which might impair muscle tissue repair (Takehara et al., 2020b). Thus, *α*-toxin is involved in disease progression *via* various mechanisms. Besides *α*-toxin, θ-toxin (or perfringlysin O), which is a pore-forming and cholesterol-dependent cytolysin, contributes to the progression of myonecrosis (Rood, 1998; Verherstraeten et al., 2015). It was reported that θ-toxin-deficient strains show delayed spread of muscle necrosis (Ellemor et al., 1999). θ-toxin also enhances intravascular cell aggregation, leading to vascular occlusion (Bryant et al., 1993; Hickey et al., 2008). Additionally, *C. perfringens* produces other toxins and enzymes including a collagenase, hyaluronidase, sialidases, and the cysteine protease *α*-clostripain (Rood, 1998; Shimizu et al., 2002). Thus, the infection process and virulence factors have become increasingly clear, but our understanding remains limited regarding the host immune exclusion mechanism after *C. perfringens* infection.

We previously showed that neutrophil elimination by anti-Ly6G treatment increases viable cell counts after injection of an *α*-toxin-deficient mutant of *C. perfringens* into skeletal muscle, suggesting that neutrophils play a key role in the elimination of *C. perfringens* (Takehara et al., 2016a). Neutrophils phagocytose and kill pathogenic bacteria and are important innate immune cells comprising the first line of host defense against invading microorganisms (Dale et al., 2008; Amulic et al., 2012; Kolaczkowska and Kubes, 2013). Generally, granulopoiesis accelerates to replenish neutrophils during bacterial infection. This so-called emergency granulopoiesis is initiated by the recognition of structural components of microorganisms; pattern recognition receptors, such as toll-like receptors (TLRs), are responsible for this recognition (Boettcher et al., 2014; Manz and Boettcher, 2014; Wirths et al., 2014). Among the TLR family members, TLR4 has been shown to recognize the Gram-negative bacterial endotoxin lipopolysaccharide (LPS) and to protect against Gram-negative bacteria (Takeuchi et al., 1999; Beutler, 2000). TLR2 has been identified as pivotal for the recognition of Gram-positive bacteria, and a cell wall component, peptidoglycan (PGN), is known to be its ligand (Takeuchi et al., 1999; Dziarski, 2003; Texereau et al., 2005). LPS or PGN stimulates TLR signaling and increases the production of granulocyte colony-stimulating factor (G-CSF), a glycoprotein that influences the proliferation, survival, and differentiation of neutrophils, leading to the acceleration of granulopoiesis (Boettcher et al., 2012; Takehara et al., 2017). Thus, TLR2 and TLR4 have been demonstrated to protect against Gram-positive or Gram-negative bacterial infection by sensing the respective structural components peculiar to each bacterium. However, the role of TLR4 in Gram-positive bacterial infection is less well understood because the bacteria do not contain LPS.

*C. perfringens* θ-toxin was demonstrated to be a TLR4 agonist (Park et al., 2004). This finding suggests that TLR4 contributes to the recognition of *C. perfringens*. Additionally, we reported that *α*-toxin amplifies LPS-induced inflammatory responses (Takehara et al., 2019a). However, the role of TLR4 in *C. perfringens* infection is unclear. In this study, we investigated whether TLR4 mutation influences susceptibility to *C. perfringens* and found that it plays an important role in elimination. Our findings provide a novel perspective for understanding the host defense mechanism against *C. perfringens*.

## Methods

### Mice

Mice aged more than 8 weeks were used for all experiments. All mice were from SLC (Shizuoka, Japan). The Animal Care and Use Committee of Tokushima Bunri University approved the animal experiments, and the procedures were performed in accordance with institutional guidelines. The institutional guidelines conform to the Fundamental Guidelines for Proper Conduct of Animal Experiment and Related Activities in Academic Research Institutions under the jurisdiction of the Ministry of Education, Culture, Sports, Science and Technology, 2006. The mice were kept in a specific pathogen-free animal facility at Tokushima Bunri University.

### Reagents and Strain

Fluorescein isothiocyanate (FITC)-, phycoerythrin (PE)- and allophycocyanin (APC)-conjugated specific antibodies against mouse CD11b (clone M1/70), Ly-6G (clone 1A8), NK1.1 (clone PK136) or CD3 epsilon (clone 145-2C11), an antibody against CD31 (clone MEC 13.3), and purified rat anti-mouse CD16/CD32 (Fc Block) were purchased from BD Biosciences (CA, USA). Antibody against interleukin-1β (IL-1β) (clone 11E5) was from Santa Cruz Biotechnology (CA, USA). Alexa Fluor 488 goat anti-rat IgG and Chromeo 546 goat anti-mouse IgG were obtained from Abcam (MA, USA). All other chemicals were of the highest grade available from commercial sources. Wild-type *C. perfringens* Strain 13 was used in this study.

### Bacterial Culture and Infection

Bacterial culture and infection were performed as previously described (Takehara et al., 2016a). *C. perfringens* strain 13 was grown in TGY (tryptone, glucose, and yeast extract) medium in anaerobic conditions at 37°C. Exponentially growing bacteria were harvested, washed, re-suspended in TGY medium, and injected into the femoral muscles of mice. Residual bacteria were serially diluted, plated on brain heart infusion agar plates, and cultured anaerobically at 37°C to quantify colony-forming units (CFUs). Survival of mice intramuscularly injected with 5 × 10^7^ CFU of *C. perfringens* was monitored.

### Immunohistochemistry

Immunohistochemistry was performed as previously described with some modifications (Takehara et al., 2019a). Mice were intramuscularly injected with 1 × 10^7^ CFU of *C. perfringens*, and *C. perfringens*-infected femoral muscles were isolated 24 h after the infection. Samples were embedded in OCT compound (Sakura Finetek Japan, Tokyo, Japan), and cryosectioning of the frozen tissue was performed using a cryostat microtome (Leica, IL, USA). Sections were blocked using Blocking One Histo (Nacalai Tesque, Inc., Kyoto, Japan), and incubated with primary antibodies. Finally, the sections were incubated for 1 h with secondary antibodies. The antibodies were diluted in DAKO Antibody Diluent (DAKO, Glostrup, Denmark). Nuclei were stained with 4′,6-diamino-2-phenylindole (DAPI). Images were captured on a confocal laser-scanning fluorescence microscope (Nikon A1, Nikon instruments, Tokyo, Japan).

### Flow Cytometry Analysis

Mice were intramuscularly injected with 1 × 10^7^ CFU of *C. perfringens*, and spleens were isolated 72 h after the infection. To isolate spleen cells, each isolated spleen was crushed in phosphate-buffered saline (PBS) supplemented with 2% heat-inactivated fetal bovine serum (FBS; AusGeneX, QLD, Australia), and filtered through a 40 μm mesh. Red blood cells were hemolyzed with lysis buffer (ACK lysing buffer; GIBCO, NY, USA). The number of living cells was counted after trypan blue staining.

Flow cytometry analysis was performed as described previously (Takehara et al., 2016a). Briefly, cells were labeled with antibodies diluted in PBS containing 2% FBS after blocking Fc-receptors with purified rat anti-mouse CD16/CD32. The labeled cells were analyzed using a Guava easyCyte (Millipore, MA, USA), and data were analyzed using FlowJo software (Tree Star, OR, USA).

### ELISA

Mice were intramuscularly injected with 1 × 10^7^ CFU of *C. perfringens*, and *C. perfringens*-infected femoral muscles were isolated 24 h after the infection. Each isolated muscle was cut into small pieces of 2–4 mm in PBS and dissociated in a gentleMACS C tube (Miltenyi Biotec, Bergisch Gladbach, Germany) using a gentleMACS dissociator (Miltenyi Biotec, Bergisch Gladbach, Germany). Measurements of G-CSF, interleukin-6 (IL-6) and IL-1β levels in the supernatant were performed using mouse Quantikine ELISA kits in accordance with the manufacturer’s instructions (R&D Systems, MN, USA).

### Quantification of *C. perfringens* CFUs in Femoral Muscle

Mice were intramuscularly injected with 1 × 10^7^ CFU of *C. perfringens*, and *C. perfringens*-infected femoral muscles were isolated 72 h after the infection. To quantify *C. perfringens* CFUs, the isolated muscles were cut into small pieces of 2–4 mm in TGY medium and dissociated in a gentleMACS C tube using a gentleMACS dissociator as described previously (Takehara et al., 2016a). The supernatants were serially diluted, plated on BHI agar plates, and cultured anaerobically at 37°C.

### Myotube Morphology Analysis

Myotube morphology was analyzed as previously described (Takehara et al., 2019b). Mice were intramuscularly injected with 1 × 10^7^ CFU of *C. perfringens*, and *C. perfringens*-infected muscles were isolated 24 h after infection. The isolated muscles were fixed in 4% paraformaldehyde and embedded in paraffin. To visualize muscle fibers, paraffin sections were cut from the tissue and stained with hematoxylin and eosin. Pictures of the muscle fibers were taken using a digital camera, and the diameters of muscle fibers were measured using DS-L4 (Nikon, Tokyo, Japan). The diameters of 100 muscle fibers were measured for each condition.

### Microarray Analysis

Mice were intramuscularly injected with 1 × 10^7^ CFU of *C. perfringens*, and *C. perfringens*-infected femoral muscles were isolated 1.5 h after the infection. The isolated muscles were cut into small pieces of 2–4 mm in lysis buffer RLT of an RNeasy mini kit (QIAGEN, Hilden, Germany) and dissociated in a gentleMACS M tube (Miltenyi Biotec, Bergisch Gladbach, Germany) using a gentleMACS dissociator. Total RNA was extracted using the RNeasy mini kit, and the quality of purified RNA was assessed by Filgen Incorporated (Aichi, Japan). Microarray analysis was also performed by Filgen Incorporated using the Clariom S Assay for mice (Thermo Fisher Scientific Incorporated, MA, USA) and GeneChip Scanner 3000 7G (Thermo Fisher Scientific Incorporated, MA, USA). Scan data were analyzed using a software package (Expression Console Software; Thermo Fisher Scientific Incorporated, MA, USA).

### Statistical Analysis

All statistical analyses were performed with Easy R (Saitama Medical Center, Jichi Medical University) (Kanda, 2013). Differences between two groups were evaluated using two-tailed Student’s *t*-test. One-way analysis of variance (ANOVA) followed by the Tukey test was used to evaluate differences among three or more groups. Differences were considered to be significant for values of P <0.05.

## Results

### TLR4 Is Involved in Host Defense Against *C. perfringens*

**Figure 1:**
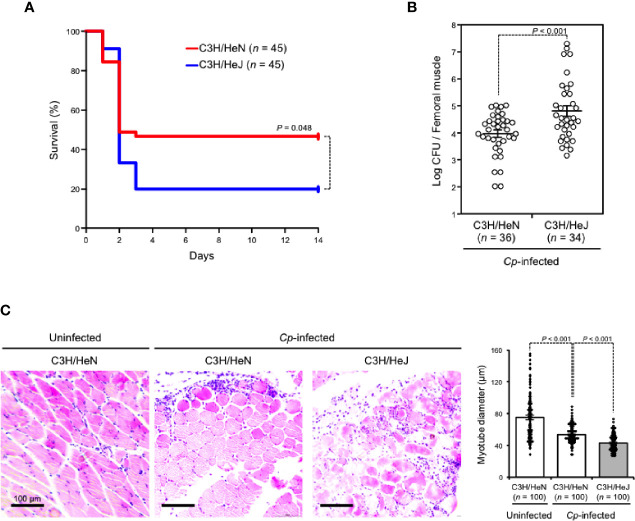
TLR4 plays an important role in effective host defense against *C*. *perfringens*. C3H/HeN and C3H/HeJ mice were intramuscularly injected with 5 × 10^7^ **(A)** or 1 × 10^7^ **(B, C)** colony-forming units (CFU) of *C*. *perfringens* Strain 13 (*Cp*-infected), or TGY medium as a control (Uninfected). **(A)** The survival of mice was monitored, and Kaplan–Meier survival curves are shown. **(B)** *C. perfringens*-infected femoral muscles were dissociated 72 h after infection, and *C*. *perfringens* CFU counts were determined. **(C)** Muscles were isolated 24 h after infection. Representative H&E-stained sections are shown, and 100 muscle fibers were measured. Log-rank test **(A)**, two-tailed Student’s t-test **(B)**, and one-way ANOVA **(C)** were employed to assess significance. Values are mean ± standard error.

C3H/HeJ mice carry a missense mutation in the third exon of the TLR4 gene causing their hyporesponsiveness to LPS, and they have been widely used to examine the role of TLR4 signaling against bacterial infections (Poltorak et al., 1998). In the present study, TLR4-defective C3H/HeJ mice and wild-type C3H/HeN mice were intramuscularly injected with *C. perfringens* type A. As shown in **Figure 1A**, about half of C3H/HeN mice (24/45) died three days after *C. perfringens* infection, whereas 80% of C3H/HeJ mice (36/45) died at the same time. To test whether this difference was caused by a difference in bacteria removal efficiency between mouse strains, the viable cell counts of *C. perfringens* in the infected muscle were measured. The numbers of viable *C. perfringens* in skeletal muscles from C3H/HeJ mice were much higher than in those from C3H/HeN mice (**Figure 1B**). Following hematoxylin and eosin (H&E staining), the severity of skeletal muscle necrosis in *C. perfringens*-infected muscle was determined. Severe edema and contraction of muscle fiber diameter were observed in C3H/HeN mice intramuscularly injected with *C. perfringens*, but the severity was greater in C3H/HeJ mice (**Figure 1C**). Taken together, TLR4 plays an important role in the elimination of *C. perfringens* and effective host defense against the bacteria.

### TLR4 Signaling Accelerates Granulopoiesis During *C. perfringens* Infection

**Figure 2:**
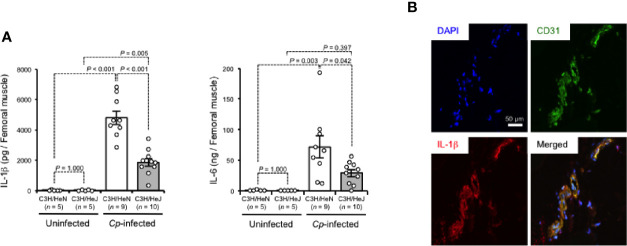
TLR4 signaling accelerates expression of inflammatory cytokines during *C*. *perfringens* infection. C3H/HeN and C3H/HeJ mice were intramuscularly injected with 1 × 10^7^ CFU of *C. perfringens* Strain 13 (*Cp*-infected), or TGY medium as a control (Uninfected). At 24 h after the infection, IL-1β and IL-6 levels in the muscle were determined **(A)**, or the muscle was subjected to immunohistochemical analysis with antibodies against CD31 and IL-1β **(B)**. One-way ANOVA was employed to assess significance. Values are mean ± standard error.

TLR4 signaling has been revealed to regulate the production of inflammatory cytokines (O’Neill, 2002). As shown in **Figure 2A**, IL-1β and IL-6 were greatly increased in *C. perfringens*-infected muscle from C3H/HeN mice, while the increases were limited in those from C3H/HeJ mice. There were no differences in the baseline expressions of these cytokines between C3H/HeJ and C3H/HeN mice (**Figure 2A**). In *C. perfringens*-infected muscle tissue from C3H/HeN mice, expression of IL-1β was detected in CD31^+^ endothelial cells (**Figure 2B**). These results suggest that TLR4 signaling is involved in the regulation of inflammatory cytokine expression during *C. perfringens* infection.

**Figure 3:**
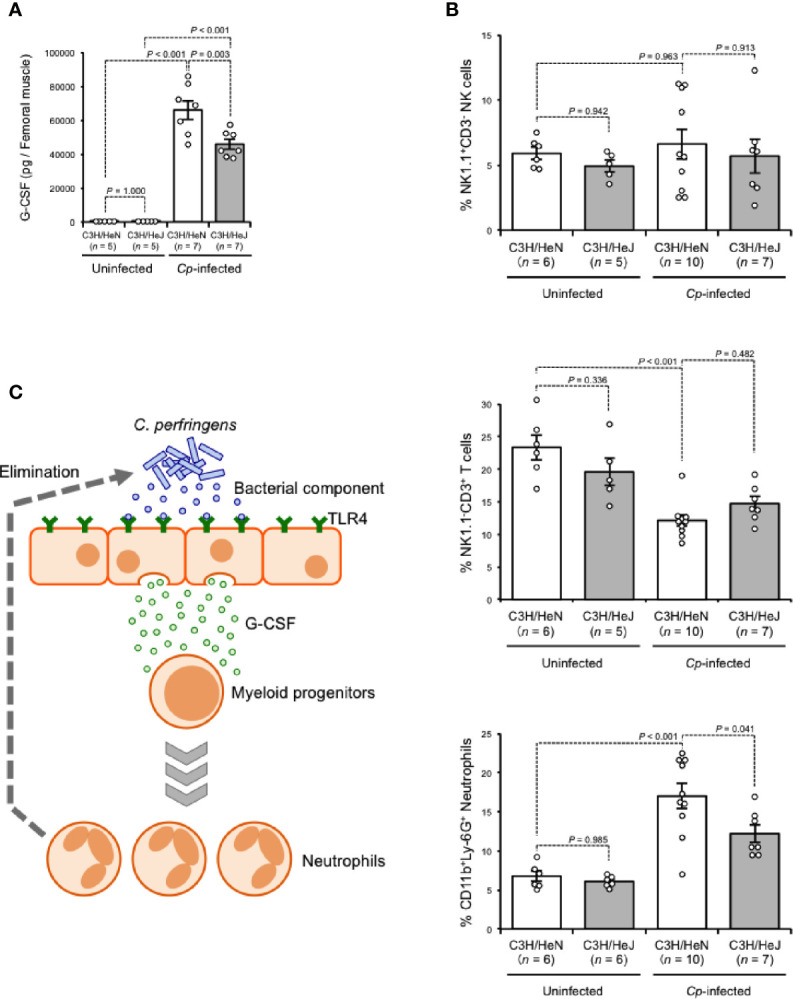
TLR4 signaling accelerates production of neutrophils. C3H/HeN and C3H/HeJ mice were intramuscularly injected with 1 × 10^7^ CFU of *C*. *perfringens* Strain 13 (*Cp*-infected), or TGY medium as a control (Uninfected). **(A)** At 24 h after infection, G-CSF levels in the muscle were determined. **(B)** At 72 h after the infection, spleen cells were isolated from the mice, and flow cytometry analysis was performed using a Guava easyCyte. The proportions of CD11b^+^Ly-6G^+^ neutrophils, NK1.1^−^CD3^+^ T cells and NK1.1^+^CD3^−^ natural killer cells are shown. **(C)** Model of accelerated production of neutrophils in *C*. *perfringens*-infected host through TLR4 signaling activation. One-way ANOVA was employed to assess significance. Values are mean ± standard error.

G-CSF is a glycoprotein that influences the proliferation, survival, and differentiation of neutrophils and their progenitor cells (Demetri and Griffin, 1991). During Gram-negative bacterial infection, LPS stimulates the production of G-CSF, leading to the acceleration of granulopoiesis, and this phenomenon depends on TLR4-expressing endothelial cells (Boettcher et al., 2014). G-CSF was greatly increased in *C. perfringens*-infected muscle from C3H/HeN mice, while the increase was limited in that from C3H/HeJ mice, suggesting that TLR4 plays a role in the regulation of granulopoiesis during *C. perfringens* infection (**Figure 3A**). During systemic *Escherichia coli* infection, stimulation of TLR4 mobilizes CD150^+^CD48^−^Lineage^−/low^Sca1^+^cKit^+^ hematopoietic stem cells (HSCs) to the spleen to give rise to neutrophils, which contributes to the host defense (Burberry et al., 2014). The number of CD11b^+^Ly-6G^+^ neutrophils was greatly increased in spleens from *C. perfringens*-infected C3H/HeN mice compared with uninfected control mice, whereas the increase was limited in *C. perfringens*-infected C3H/HeJ mice (**Figure 3B**). Meanwhile, the numbers of NK1.1^−^CD3^+^ T cells and NK1.1^+^CD3^−^ natural killer cells were similar in both mouse lines (**Figure 3B**). Together, our results indicate that TLR4 signaling plays an important role in the regulation of G-CSF-mediating granulopoiesis during *C. perfringens* infection, and this mechanism probably contributes to the replenishment of neutrophils and the elimination of bacteria (**Figure 3C**).

### TLR4 Signaling Is Partially Involved in Changes in Host Gene Expression Associated With *C. perfringens* Infection

**Figure 4:**
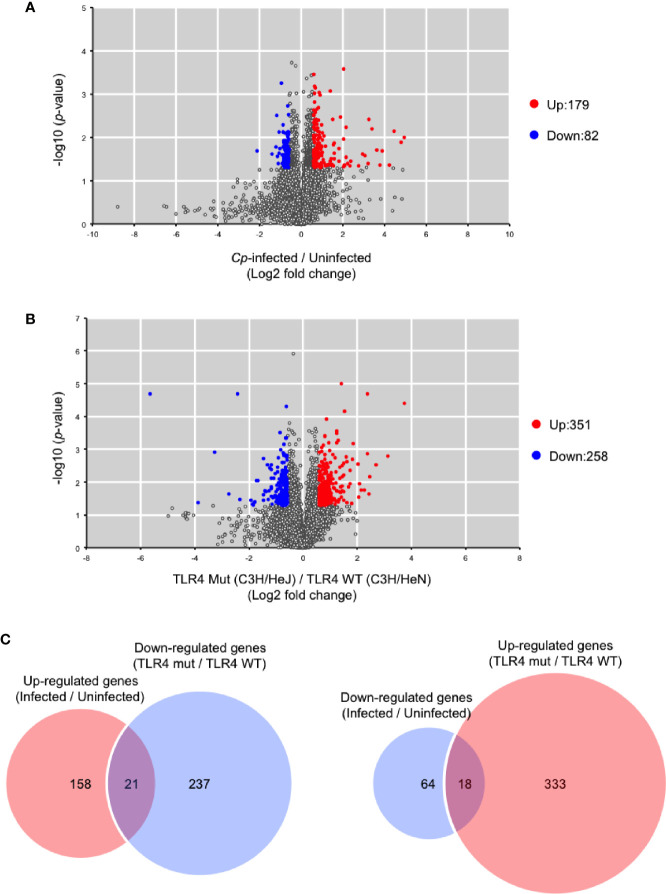
Differentially expressed genes between C3H/HeN and C3H/HeJ mice during *C*. *perfringens* infection. C3H/HeN and C3H/HeJ mice were intramuscularly injected with 1 × 10^7^ CFU of *C. perfringens* Strain 13 (*Cp*-infected), or TGY medium as a control (Uninfected). At 1.5 h after infection, muscles were isolated from the mice, and total RNAs from the tissues were subjected to DNA microarray analysis as described in *Methods*. **(A)** Volcano plot depicting information about differentially expressed genes between *C*. *perfringens*-infected and uninfected muscles from C3H/HeN mice. **(B)** Volcano plot depicting information about differentially expressed genes in *C*. *perfringens*-infected muscles between C3H/HeN and C3H/HeJ mice. **(C)** Venn diagrams for microarray data.

It was previously demonstrated that 1,055 host genes are upregulated, and 386 host genes are downregulated in response to *C. perfringens* infection by RNA sequencing (Low et al., 2018). In that study, RNA was extracted from mice at 1.5 h post-infection because disease progression in murine models is rapid. In the present study, RNA was also extracted from *C. perfringens*-infected muscle at 1.5 h post-infection, and host gene expression was measured by DNA microarray analysis. In *C. perfringens*-infected C3H/HeN mice, 179 genes were upregulated more than 1.5-fold, and 82 genes were downregulated less than 1.5-fold compared with uninfected control mice (**Figure 4A**). Host immunity genes, such as *CXCL2*, *Trem1*, *CXCL1*, *Il1b*, and *CXCL3*, were significantly upregulated in *C. perfringens*-infected mice (**Supplementary Table S1**). These genes were already reported to be upregulated in *C. perfringens*-infected mice, so our results corroborate the previous report (Low et al., 2018). Next, a comparison of gene expression levels between C3H/HeN and C3H/HeJ mice was performed. In *C. perfringens*-infected C3H/HeJ mice, 351 genes were upregulated more than 1.5-fold, and 258 genes were downregulated less than 1.5-fold compared with *C. perfringens*-infected C3H/HeN mice (**Figure 4B**). The genes are listed in **Supplementary Table S2**. Among the 179 genes upregulated by *C. perfringens* infection, 21 genes were downregulated in C3H/HeJ mice compared with C3H/HeN mice (**Figure 4C**). Also, 18 genes were upregulated in C3H/HeJ mice compared with C3H/HeN mice among the 82 genes downregulated by *C. perfringens* infection (**Figure 4C**). The genes are listed in **Supplementary Table S3**. These results demonstrate that TLR4 signaling is partially involved in changes in host gene expression associated with *C. perfringens* infection.

## Discussion

*C. perfringens* type A is a Gram-positive bacterium that causes gas gangrene characterized by severe myonecrosis (Songer, 1996; Petit et al., 1999). It has been demonstrated using TLR2-deficient mice that TLR2 contributes to the host defense against Gram-positive bacterial infection. TLR2-deficient mice are greatly susceptible to *Staphylococcus aureus*, showing impaired clearance of the infected bacteria (Takeuchi et al., 2000; Hoebe et al., 2005). Similarly, TLR2-deficiency reduces the elimination of *Streptococcus pneumoniae*, resulting in increased susceptibility to the bacteria (Echchannaoui et al., 2002). In the clinical setting, some polymorphisms in the *Tlr2* gene, which attenuates TLR2 signaling, increase the risk of severe tuberculosis caused by *Mycobacterium tuberculosis* (Ben-Ali et al., 2004; Ogus et al., 2004). Moreover, TLR2 polymorphisms affect immune responses to *Mycobacterium leprae* (Kang et al., 2002; Bochud et al., 2003). Thus, TLR2 has been demonstrated to play an important role in the recognition and elimination of Gram-positive bacteria. However, the role of TLR4 in Gram-positive bacterial infection is less well understood. The present study found that TLR4-defective C3H/HeJ mice infected with *C. perfringens* have a remarkably lower survival rate, an increase in viable bacterial counts, and accelerated destruction of myofibrils at the infection site compared with wild-type C3H/HeN mice. These results indicate that TLR4 plays an important role in the elimination of *C. perfringens* and the host defense against the bacteria. Similar to our results, TLR4 was shown to contribute to host protection against *M. tuberculosis* infection (Park et al., 2020). Thus, it is important to pay attention to the function of TLR4 during Gram-positive bacterial infection.

Gram-positive bacteria do not contain LPS, but some ligands of TLR4 have been identified from these bacteria. Within the components of *M. tuberculosis*, lipomannans and heat shock proteins bind to TLR4 and activate immune cells (Hossain and Norazmi, 2013). It was also reported that *M. tuberculosis* itself binds to TLR4 (Sepehri et al., 2019). Anthrolysin O (ALO) from *Bacillus anthracis* can activate TLR4, inducing an apoptotic response in bone marrow-derived macrophages (BMDMs) (Park et al., 2004). ALO is a cholesterol-dependent cytolysin (CDC), and the other CDCs, such as *C. perfringens* θ-toxin, *Listeria monocytogenes* listeriolysin O, and *Streptococcus pyogenes* streptolysin O, can activate BMDMs through TLR4 signaling (Park et al., 2004). Thus, some Gram-positive bacteria contain TLR4 ligands, and it might be possible that activation of TLR4 at the time of Gram-positive bacterial infection is a common phenomenon.

Pattern recognition receptors, such as TLRs, are responsible for the recognition of structural components of microorganisms, and their activation affects the expression of inflammatory cytokines (O’Neill, 2002). Among the TLR family members, TLR4 has been shown to recognize the Gram-negative bacterial endotoxin LPS, and TLR2 has been identified as pivotal for the recognition of cell wall components such as PGN (Takeuchi et al., 1999; Beutler, 2000; Dziarski, 2003; Texereau et al., 2005). During Gram-negative bacterial infection, LPS stimulates the production of G-CSF, which is a glycoprotein, leading to the acceleration of granulopoiesis, and this phenomenon depends on TLR4-expressing endothelial cells (Boettcher et al., 2014). Similarly, PGN is suggested to stimulate the production of G-CSF in TLR2-expressing endothelial cells (Takehara et al., 2017). G-CSF promotes the proliferation and differentiation of neutrophils and their progenitor cells (Demetri and Griffin, 1991). G-CSF-deficient mice exhibit chronic neutropenia and reduced infection-driven granulopoiesis. Granulopoiesis is also impaired in G-CSF receptor-deficient mice (Lieschke et al., 1994). Thus, G-CSF plays a key role in regulating granulopoiesis during bacterial infection, and TLR2 and TLR4 are essential to the host defense mechanism. The present study observed remarkable increases in the levels of inflammatory cytokines IL-1β, IL-6, and G-CSF, in *C. perfringens*-infected C3H/HeN mice. The cytokines would be secreted from endothelial cells but not infiltrating immune cells because it was demonstrated that the infiltration of immune cells is impaired by *α*-toxin in *C. perfringens*-infected muscle (Takehara et al., 2016a). The result shown in **Figure 2B** reinforces this idea. Moreover, the cytokines were only partially increased in C3H/HeJ mice. The results suggest that TLR2 plays a complementary role to TLR4 during *C. perfringens* infection. It might be that TLR2 and TLR4, signaling cooperatively, regulate the production of G-CSF to control granulopoiesis effectively and strictly. Otherwise, further studies will be needed to unveil the functional difference between TLR2 and TLR4 signaling on the regulation of neutrophil production.

It has been reported that activation of TLRs should be properly regulated to avoid tissue damage by excessive inflammation (Kawai and Akira, 2010). Ubiquitin ligases, which are splice variants for adaptors and transcriptional regulators, have been identified as negative regulators of TLR signaling (Kawai and Akira, 2010). We recently reported that *α*-toxin amplifies LPS-induced inflammatory responses and increases the lethal toxicity of LPS, which is dependent on TLR4 (Takehara et al., 2019a). In the previous report, it was unclear whether it is meaningful to speculate on the role of α-toxin in the TLR4-mediated inflammatory response in *C. perfringens* infection, because *C. perfringens* do not contain LPS. In the present study, we showed that TLR4 plays an important role in the elimination of *C. perfringens*, indicating that TLR4 signaling is activated during infection. As described above, θ-toxin was reported to be a TLR4 agonist (Park et al., 2004). Together, it might be possible that θ-toxin activates TLR4, and that the excessive activation of TLR4 by *α*-toxin contributes to the characteristics of *C. perfringens* infection, such as the destruction of muscle, shock, multiple organ failure, systemic inflammation, and death of patients. In conclusion, our results indicate that TLR4 plays an important role in the elimination of *C. perfringens*, which provides a novel perspective for understanding the pathogenesis of *C. perfringens*.

## Data Availability Statement

The raw data supporting the conclusions of this article will be made available by the authors, without undue reservation.

## Ethics Statement

The animal study was reviewed and approved by Animal Care and Use Committee of Tokushima Bunri University.

## Author Contributions

MT and MN designed the study and supervised experiments. MT performed experiments and analyses, and wrote the manuscript. KK contributed to the design of *in vivo* studies. All authors contributed to the article and approved the submitted version.

## Funding

This work was supported by a Grant-in-Aid for Scientific Research from the Ministry of Education, Culture, Sports, Science, and Technology of Japan (grant number 18K07129).

## Conflict of Interest

The authors declare that the research was conducted in the absence of any commercial or financial relationships that could be construed as a potential conflict of interest.
